# Evaluation of a melanocortin-4 receptor (MC4R) agonist (Setmelanotide) in MC4R deficiency

**DOI:** 10.1016/j.molmet.2017.06.015

**Published:** 2017-07-08

**Authors:** Tinh-Hai Collet, Béatrice Dubern, Jacek Mokrosinski, Hillori Connors, Julia M. Keogh, Edson Mendes de Oliveira, Elana Henning, Christine Poitou-Bernert, Jean-Michel Oppert, Patrick Tounian, Florence Marchelli, Rohia Alili, Johanne Le Beyec, Dominique Pépin, Jean-Marc Lacorte, Andrew Gottesdiener, Rebecca Bounds, Shubh Sharma, Cathy Folster, Bart Henderson, Stephen O'Rahilly, Elizabeth Stoner, Keith Gottesdiener, Brandon L. Panaro, Roger D. Cone, Karine Clément, I. Sadaf Farooqi, Lex H.T. Van der Ploeg

**Affiliations:** 1 University of Cambridge Metabolic Research Laboratories and NIHR Cambridge Biomedical Research Centre, Wellcome Trust-MRC Institute of Metabolic Science, Box 289, Addenbrooke's Hospital, Hills Road, Cambridge, CB2 0QQ, United Kingdom; 2 Service of Endocrinology, Diabetes and Metabolism, University Hospital of Lausanne, Lausanne, 1011, Switzerland; 3 Institute of Cardiometabolism and Nutrition (ICAN), Assistance Publique-Hôpitaux de Paris, Trousseau and Pitié-Salpêtrière Hospitals, F-75013, Paris, France; 4 Sorbonne Université, UPMC Univ Paris 06, UMR_S 1166, INSERM, F-75013, Paris, France; 5 Rhythm Pharmaceuticals, 500 Boylston Street, Boston, MA, 02116, USA; 6 Assistance Publique, Hôpital Pitié-Salpêtrière-Charles Foix, Department of Endocrine and Oncological Biochemistry, F-75651, Paris, France; 7 Sorbonne Université, UPMC Univ, Paris 06, France; 8 Inserm UMR1149, F-75890, Paris, France; 9 Lunenfeld-Tanenbaum Research Institute, Department of Medicine, Mt. Sinai Hospital, University of Toronto, Toronto, Ontario, Canada; 10 Vanderbilt University, Department of Molecular Physiology and Biophysics, Nashville, TN, 37235, USA; 11 Life Sciences Institute and Department of Molecular and Integrative Physiology, University of Michigan, Ann Arbor, MI, 48105, USA

**Keywords:** Obesity, Melanocortin 4 receptor, Setmelanotide, Stratification

## Abstract

**Objective:**

Pro-opiomelanocortin (POMC)-derived peptides act on neurons expressing the Melanocortin 4 receptor (MC4R) to reduce body weight. Setmelanotide is a highly potent MC4R agonist that leads to weight loss in diet-induced obese animals and in obese individuals with complete POMC deficiency. While POMC deficiency is very rare, 1–5% of severely obese individuals harbor heterozygous mutations in *MC4R*. We sought to assess the efficacy of Setmelanotide in human MC4R deficiency.

**Methods:**

We studied the effects of Setmelanotide on mutant MC4Rs in cells and the weight loss response to Setmelanotide administration in rodent studies and a human clinical trial. We annotated the functional status of 369 published *MC4R* variants.

**Results:**

In cells, we showed that Setmelanotide is significantly more potent at MC4R than the endogenous ligand alpha-melanocyte stimulating hormone and can disproportionally rescue signaling by a subset of severely impaired MC4R mutants. Wild-type rodents appear more sensitive to Setmelanotide when compared to MC4R heterozygous deficient mice, while MC4R knockout mice fail to respond. In a 28-day Phase 1b clinical trial, Setmelanotide led to weight loss in obese *MC4R* variant carriers. Patients with POMC defects upstream of MC4R show significantly more weight loss with Setmelanotide than MC4R deficient patients or obese controls.

**Conclusions:**

Setmelanotide led to weight loss in obese people with MC4R deficiency; however, further studies are justified to establish whether Setmelanotide can elicit clinically meaningful weight loss in a subset of the MC4R deficient obese population.

## 1 Introduction

Neural circuits in the hypothalamus play a critical role in the regulation of energy homeostasis [1], [2]. Peripheral nutrient-derived signals such as leptin stimulate the expression of Pro-opiomelanocortin (POMC)-derived melanocortin peptides, which reduce food intake and body weight by acting as endogenous ligands for the brain-expressed melanocortin-4 receptor (MC4R) [3], [4]. *MC4R* knockout mice are severely obese while loss of one *MC4R* allele results in an intermediate obesity phenotype demonstrating that weight regulation is sensitive to quantitative variation in MC4R expression [5]. Obesity-associated *MC4R* mutations confirm a role for MC4R-mediated signaling in human energy homeostasis [6], [7], [8], [9], [10]. Additionally, MC4R activation may be modulated by accessory proteins such as melanocortin receptor accessory protein 2 (MRAP2) [11], [12], [13].

In view of these effects, MC4R, which is a seven-transmembrane domain G-protein coupled receptor (GPCR), has been considered as a potential drug target for the treatment of obesity [14], [15], [16]. However, first generation MC4R agonists resulted not only in suppression of food intake and induction of weight loss, but also in a significant increase in blood pressure (BP) and heart rate (HR) in rodents, primates, and humans [16], [17], [18], [19]. For example, treatment of obese volunteers with the agonist LY2112688 at the maximum dose of 1.0 mg/day led to significant increases of systolic (mean 9.3 ± SD 1.9 mmHg) and diastolic (mean 6.6 ± SD 1.1 mmHg) blood pressure after only 24 h of treatment compared with placebo [18]. These adverse effects halted the development of the first generation of MC4R agonists.

Setmelanotide (formerly known as RM-493 or BIM-22493) is a synthetic cyclic peptide that binds to human MC4R with high affinity, activates MC4R at nanomolar concentrations (50% effective concentration [EC_50_] = 0.27 nM), and has been shown to reduce food intake and body weight and restore insulin sensitivity in diet-induced obese (DIO) mice, rats, dogs, and monkeys [20], [21]. Comparable weight loss effects have been observed in genetic animal models of obesity treated with Setmelanotide, although not in *MC4R* knockout mice [22], indicating that the effects of Setmelanotide are indeed due to its action on MC4R. Importantly, in Rhesus monkeys with diet-induced obesity, Setmelanotide infused at 0.50 mg/kg/day for 8 weeks led to significant decreases in food intake (∼40%) and body weight (∼13%) without acute or long-term increases in BP or HR measured by continuous telemetry [20]. Additionally, drug-related changes in heart rate and blood pressure were not observed in a series of Setmelanotide phase 1b/2a or experimental studies in obese individuals [21].

In a recent proof-of-principle study, two rare POMC deficient individuals treated with Setmelanotide experienced significant weight loss (51.0 kg of body weight loss after 42 weeks in patient 1 and 20.5 kg of body weight after 12 weeks in a second patient) without any adverse effects on cardiovascular parameters [23]. These results established that while obesity due to complete POMC deficiency is very rare, it is potentially very responsive to treatment with Setmelanotide. Heterozygous loss of function (LOF) mutations in *MC4R* are found more frequently with prevalence estimates ranging from 1 to 5% in children or adults with severe obesity [9], [10], [24]. This group of individuals are relatively refractory to weight loss through diet and exercise [25] and other treatment options remain limited. Roux-en-Y bypass surgery can be effective, but is not without risks and not suitable for many people [26]. We hypothesized that Setmelanotide might lead to weight loss in MC4R deficiency by rescuing signaling by mutant MC4Rs and/or by increasing signaling through the wild type MC4R allele. We therefore performed a series of studies, which included a detailed interpretation of the functional status of 369 MC4R variants as well as preclinical and clinical studies in a small subset of variants to address the impact of MC4R mutations or Setmelanotide treatment on energy homeostasis in MC4R deficiency.

## 2 Materials and methods

### 2.1 Cohorts

UK: Ethical approval for studies was given by the Cambridge Research Ethics Committee; all participants gave written informed consent. The Genetics of Obesity Study (GOOS) is a cohort of 7000 individuals with severe early-onset obesity; age of obesity onset is less than 10 years. Severe obesity is defined as a body mass index (BMI, weight in kilograms divided by the square of the height in meters) standard deviation score ≥ 3 (SDS, calculated according to the United Kingdom reference population). *MC4R* sequencing was performed as described previously.

France: Subjects were patients followed-up in the Department of Nutrition of Trousseau hospital (for children), Hôtel-Dieu and Pitié-Salpêtrière hospital for adults (Paris, France). They were part of a genetic study of severe obesity (Clinical Research program PHRC 0702) with similar inclusion criteria of the UK cohort. Clinical investigations were approved by the Ethics Committee of Hôtel-Dieu hospital. All subjects gave their written informed consent. *MC4R* sequencing was performed at UF Genetic of Obesity and Dyslipidemia of Hôtel-Dieu and Pitié-Salpêtrière hospitals (J.L.B., D.P., J.M.L.).

### 2.2 Systematic review of literature on MC4R variants

To assess the functional consequences of all published MC4R mutations, we searched all studies on functional cell-based assays of MC4R variants. We systematically reviewed articles containing the terms “MC4R” or “mc4-r” indexed on MEDLINE from inception to August 31st, 2016 in addition to bibliographies of manually selected articles, mutations published by human genetic databases (1000 Genomes release 16, Exome Variant Server ESP6500SI-V2) and unpublished data from our labs. Because functional consequences of MC4R mutations are assessed and reported in a wide variety of ways, we devised an *a priori* semi-quantitative scale of the overall residual function. Each non-synonymous mutation could lead to a complete LOF, a partial LOF, or no LOF (wild type like). In addition, contradictory and unclear published data resulted in ambiguous status for many variants. Functional assay data were collected for each published MC4R variant: cell line, cell surface expression, tested ligands (α-MSH, β-MSH, γ1-MSH and artificial agonists such as NDP-α-MSH, melanotan [MT-II]), E_max_ (efficacy, the maximum level of cAMP production in response to increased doses of a given ligand), EC_50_ (potency, the concentration of a ligand eliciting half of the maximum response in cAMP production), and in a competitive binding assay B_max_ (the maximal binding capacity to a ligand) and IC_50_ (affinity, the concentration of a ligand binding half the receptors). All functional data were normalized to wild type levels, to enable comparison between articles (Figures S1–S2). The assay data were independently abstracted by three authors (T.-H.C., B.D., A.G.) and compared to reach a consensus on the residual function of each variant. Discrepancies were discussed among co-authors to reach a consensus on the residual function of each MC4R variant. Ambiguous variants were re-evaluated in a head-to-head comparison of activity in cell-based assays (see section Functional characterization of additional MC4R variants).

### 2.3 Comparison of response to alpha-MSH and Setmelanotide in cell-based assays

Setmelanotide (formerly known as RM-493 or BIM-22493) is a synthetic 8 amino-acid, cyclic peptide MC4R agonist with a molecular formula of C_49_H_68_N_18_O_9_S_2_ (anhydrous free base) and molecular weight of 1117.3 Da. Setmelanotide binds with high affinity to the human MC4R (inhibitory constant [Ki] = 2.1 nmol/l) and is efficient in activating MC4R (EC_50_ = 0.27 nmol/l) [20]. Activation of other melanocortin receptors by Setmelanotide requires concentrations that are more than 20-fold or higher than for MC4R: the EC_50_ was 5.8 nmol/l with MC1R, 5.3 nmol/l with MC3R, and >1 μmol/l for MC5R. Setmelanotide displayed no activity for the MC2R, also known as the adrenocorticotropic hormone (ACTH) receptor. This compound was initially selected for clinical development based on the extended circulating half-life in non-human primates (2.8–3.5 h). For the data shown in Figure 2, MC4R wild type and mutant cDNAs were cloned into DiscoverX expression vectors (RPN-MC4R (variant)-PK 7293 bp). The sequences of the inserts were fully verified and confirmed to be 100% identical to the expected nucleotide sequences. The receptors were introduced into U2OS cells expressing arrestin-AE. Cells were placed under antibiotic selection to ensure stable integration of the fusion gene construct. Expression level of each of the mutants and wild type proteins was tested. The stably transfected cell pools were plated for agonist measurements in the DiscoverX cAMP and PathHunter arrestin assays.

### 2.4 Functional characterization of MC4R variants in cell-based assays

In order to characterize newly identified MC4R variants or to clarify classification of variants for which the systematic review of published data returned ambiguous results, we performed *in vitro* assays in transiently transfected HEK293 cells. We assessed relative cell surface expression of the mutant receptors in cell-based ELISA and we employed the cAMP response element-binding protein (CREB) trans-reporting system (Stratagene) to examine the impact of these variants on the receptor signaling activity via cAMP dependent pathway. MC4R cDNA constructs barring an N-terminal FLAG tag in pCDNA3.1(+) vector (Invitrogen) were used throughout the study. Site-directed mutagenesis was performed using QuikChange kit (Agilent) according to the manufacturer's protocols. All constructs were verified with Sanger sequencing. HEK293 cells were maintained in high glucose Dulbecco's modified eagle medium (DMEM, Gibco, 31966) supplemented with 10% fetal bovine serum (Gibco, 10270, South America origin), 1% GlutaMAX™ (100X) (Gibco, 35050), 100 units/mL penicillin, and 100 μg/mL streptomycin (Sigma–Aldrich, P0781). Cells were incubated at 37 Celsius degrees in humidified air containing 5% CO_2_ and transfections were performed using Lipofectamine 2000™ (Gibco, 11668) in serum-free Opti-MEM I medium (Gibco, 31985) according to the manufacturer's protocols. Briefly, 40,000 cells/well were seeded in a poly-d-lysine coated 96-well plate and transfected the next day with either 30 or 5 ng of MC4R constructs, for cell surface expression ELISA and CREB reporter assay, respectively.

#### 2.4.1 Cell surface expression

Cells transfected with FLAG-tagged MC4R constructs were tested for cell surface localization of the receptor with ELISA. Two days after transfection, cells were fixed with 3.7% paraformaldehyde for 15 min at room temperature and washed three times with PBS. Subsequently, non-specific binding sites were blocked with 3% non-fat dry milk in 50 mM Tris-PBS pH 7.4 (blocking buffer) for 1 h at room temperature. Next, cells were incubated with a mouse monoclonal anti-FLAG (M2) antibody (Sigma–Aldrich, F1804) (dilution 1:1000 in blocking buffer) overnight at 4 Celsius degrees followed by triple washing with PBS and incubation with goat anti-mouse IgG(H + L)-HRP conjugate (Bio-Rad Laboratories, 172-1011) (1:1250 in 1.5% non-fat dry milk in 50 mM Tris-PBS pH 7.4) for 2 h at room temperature. Finally, plates were washed three times with PBS and a high performance chromogenic substrate – 3,3′,5,5′ tetramethylbenzidine (TMB CORE+, Bio-Rad Laboratories, BUF062) was used to detect HRP activity. The reaction was terminated with 0.2M H_2_SO_4_ and absorbance by the color reaction product at 450 nm was determined using Infinite M1000 PRO microplate reader (Tecan).

#### 2.4.2 CREB-luciferase reporter assay

In order to detect MC4R signaling activity, HEK293 cells were transiently co-transfected with a respective MC4R construct, a trans-activator plasmid pFA2-CREB and a reporter plasmid, pFR-Luc at 5, 6 and 25 ng/well, respectively. On the following day, cells were stimulated with increasing concentration of α-MSH for 5 h. The CREB-luciferase activity was determined using Steadylite plus substrate (Perkin Elmer, 6066759). Briefly, cells were washed with Dulbecco's modified PBS (DPBS) supplemented with 0.9 mM CaCl_2_ and 0.5 mM MgCl_2_ (Gibco, 14040). DPBS and ready-to-use luciferase substrate were dispensed in equal volumes, and luminescence was quantified after 20 min using TopCount Microplate Counter (Packard). All results were analyzed using GraphPad Prism 6 (GraphPad Software). Relative cell surface expression is presented as means ± SEM of three to four independent experiments, each normalized to basal signal for mock transfected cells and MC4R WT expression level in a given assay. CREB-luciferase reporter assays were carried out in triplicates and were independently replicated at least three times. Results of each experiment were normalized to basal signal for mock transfected cells and maximal efficacy of α-MSH for MC4R WT in a given assay. Presented dose–response curves were plotted from the merged normalized data analyzed with 4-parameter non-linear regression equation.

### 2.5 Setmelanotide administration in animals

For the duration of the Setmelanotide studies, rodents were single-housed in an IACUC approved facility at Vanderbilt University. Wildtype (*Mc4r* ^+/+^), heterozygous (*Mc4r* ^+/−^) and MC4R null (*Mc4r* ^−/−^) mice were placed on a high fat diet (Research Diets, Inc. #D12451, 45% kcal fat, 35% kcal carb, 20% kcal protein) at 11 weeks of age, which continued for the duration of the experiment. At 13 weeks of age, the animals were placed in a Promethion Sable monitoring system for 3 days to acclimatize and to obtain baseline measurements. After the acclimatization period, animals were weighed and surgically implanted with primed Alzet minipumps (Alzet #1002, 0.25 μL/h infusion rate) delivering saline alone, or 1200 nmol/kg/day of Setmelanotide dissolved in saline. The pumps were placed subcutaneously on each animal's upper back. Animals were monitored for food and water intake, then removed from the Promethion cages, and weighed once more 9 days after the implant. Statistical analyses were performed using Graphpad Prism 5.

### 2.6 Setmelanotide clinical trial

The clinical trial protocol was reviewed and approved by Schulman Associates Institutional Review Board, 4445 Lake Forest Drive, Suite 300 Cincinnati, OH 45242, USA. All clinical trial participants provided informed consent prior to enrolling in the study (ClinicalTrials.gov Identifier: NCT02431442).

*Weight*: All measurements were performed in triplicate throughout the study. The same scale was used throughout the study, including the screening visit, and was calibrated daily. Weight was measured when individuals were fasted at approximately the same time each day. Individuals were in scrubs, with no shoes and an empty bladder. Individuals were blinded to their weight throughout the study.

*Waist circumference*: All measurements were performed in triplicate. Measurements occurred during screening, at baseline, once weekly during treatment, and at the follow-up visits.

*Vital signs*: Blood pressure (BP) and heart rate (HR) were obtained in the supine position following at least 5 min of rest each time they were measured. Pre-dose measurements were taken in triplicate, approximately 2 min apart; the means of these triplicate measurements served as the baseline values. All other BP and HR time-points were single measurements. When possible, BP was taken in the same arm as it was taken at pre-dose baseline. For the first day, vitals were taken every hour for the first 12 h, then every 2 h. On Day 2, vitals were taken every 12 h, followed by once daily thereafter. On days 8, 14, 22, and 28, vitals were taken every 12 h.

Ambulatory BP monitoring (ABPM): Equipment and software was provided by CoreLab Partners, Rockville, MD 20850, USA. The ABPM monitor was worn continuously for a minimum of 24 h at 3 timepoints throughout the study: baseline, approximately mid-way through dosing duration, and at the end of dosing. The ABPM device was programmed to take readings every 20 min during the 24-hour monitoring period.

*Quantitative skin color measurements*: Quantitative skin measurements were performed with a spectrophotometric device, the Mexameter-18, Courage + Khazaka Electronic, GmbH, Cologne, Germany. The amount of melanin in the skin was measured at the 3 following locations representing various degrees of sun exposure for a total of 3 times each and the values were averaged: On the cheek of the subject, directly under the pupil on the zygomatic arch, on the forehead of the subject, midline about an inch above eyebrows, and on the upper, outer, right buttock (‘hip’). Measurements were taken prior to dosing, weekly during dosing, and at follow-up visits.

## 3 Results

### 3.1 A single system for functional annotation of MC4R mutations

**Figure 1:**
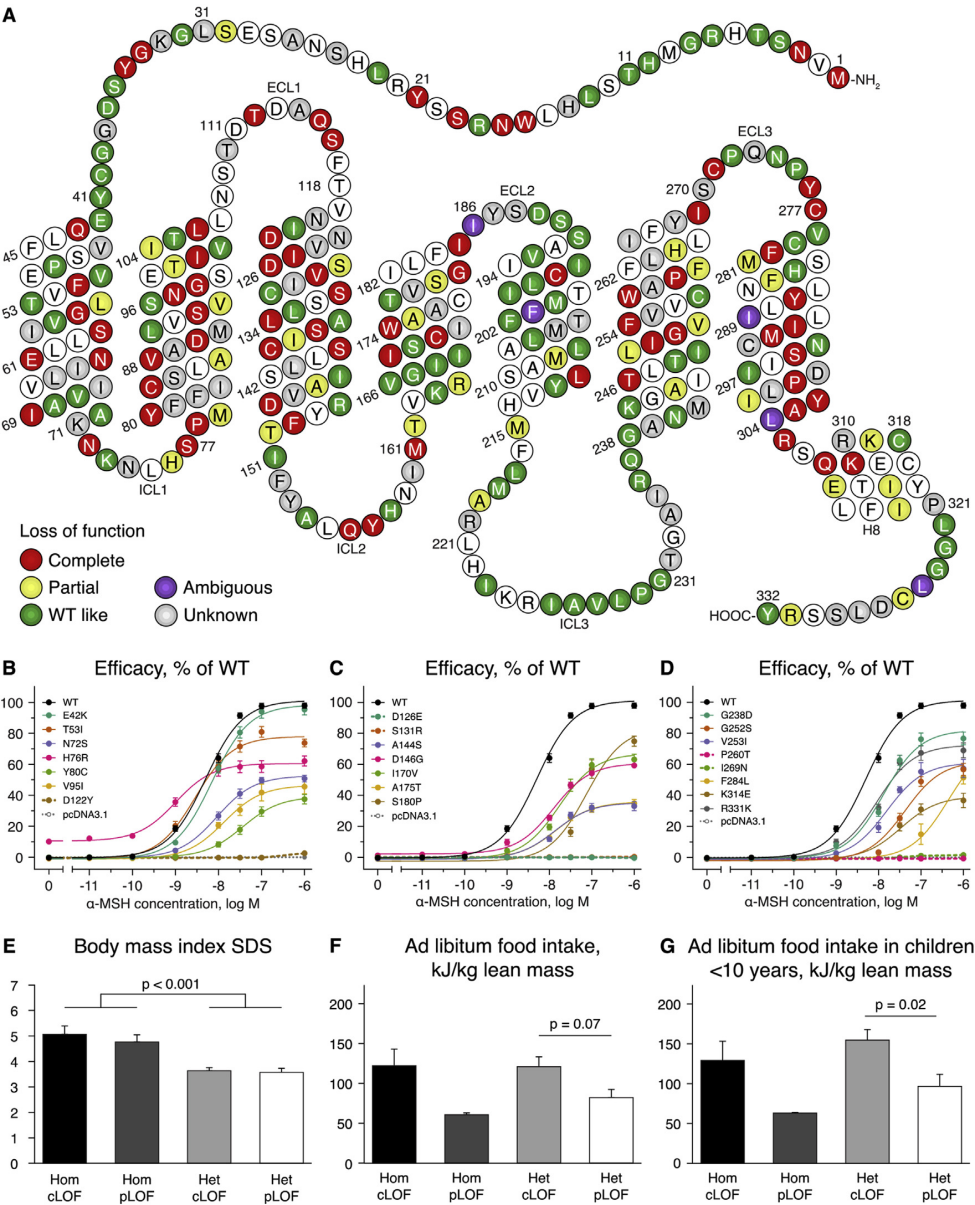
**Genetic and molecular classification of MC4R mutations.** (A) MC4R is a seven transmembrane domain GPCR with 3 intracellular loops (ICL), 3 extracellular loops (ECL), and a C-terminal helix 8 (H8); schematic adapted from GPCRDB Tools [41]. Colors denote amino acids that have been altered by mutations. Functional consequences of these mutations were classified following a systematic review of the literature (Figure S1). Where multiple variants affecting the same amino acid have been reported, the most severe loss of function (LOF) mutation is shown. (B–D) Functional characterization of 22 new MC4R mutations. α-MSH stimulated cAMP generation was assessed in transiently transfected cells using a CREB-luciferase reporter assay (Table S1). Data are mean ± SEM of 4 experiments. (E–G). Genotype-phenotype correlations in 249 children with complete or partial loss of function MC4R mutations (cLOF/pLOF respectively). Data are represented as mean ± SEM. (E) Body mass index (BMI) standard deviation scores (SDS) of children who were homozygous (Hom) for cLOF (n = 16) or pLOF mutations (n = 17) were compared to those who were heterozygous (Het) for cLOF (n = 131) or pLOF (n = 85) MC4R mutations. Homozygotes had a significantly higher BMI SDS than those with heterozygous MC4R mutations (p < 0.001 with and without adjustment for age, sex and ethnicity), while there was no statistically significant difference between those with cLOF or pLOF mutations within the same gene dosage group (both p = 1.00). (F) *Ad libitum* food intake adjusted for lean mass measured by dual energy X-ray absorptiometry in 57 children (age < 18 years) (Hom cLOF (n = 6); Hom pLOF (n = 3); Het cLOF (n = 29); Het pLOF (n = 19)). (G) *Ad libitum* food intake adjusted for lean mass in 35 children age < 10 years (Hom cLOF (n = 5); Hom pLOF (n = 2); Het cLOF (n = 17); Het pLOF (n = 11)) (data on obese children have been reported previously [10].

We generated a single functional classification system for variants in *MC4R* as not all variants impact MC4R signaling in cell based assays and this has caused some confusion in the field. Moreover, while some classification schemes have been proposed [14], [27], [28], [29], these remain difficult to apply uniformly given the discordant and/or incomplete functional data available in different publications. We systematically reviewed and collated data on all published *MC4R* variants and included unpublished data on 75 variants from our labs (Figure 1A, Figures S1–2). In total, we identified missense (n = 318, 69.7%), nonsense (n = 24; 5.3%), and frameshift mutations (n = 21; 4.6%) and deletions (n = 6; 1.3%). There were inconsistencies between the reported functional consequences of a subset of variants. To enable reliable classification of these variants, we performed functional assays on 22 *MC4R* variants with unknown, incomplete or ambiguous functional status for which we had clinical data; 5 resulted in a complete LOF, 14 in a partial LOF and 3 were wild type (WT)-like (Figure 1B–D, Table S1). We have made the functional classification dataset with MC4R functional assay data, available as a searchable resource (Table S2, www.mc4r.org.uk).

**Figure 2:**
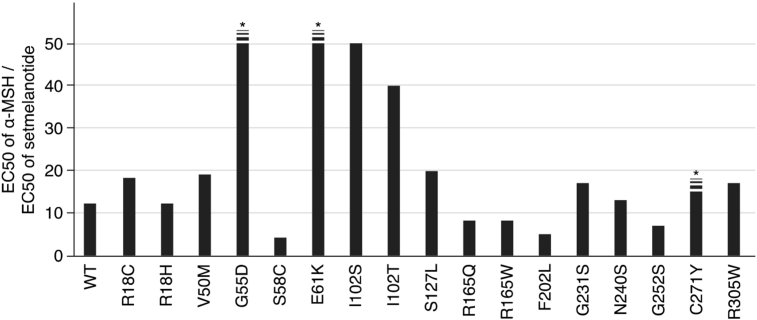
Effects of Setmelanotide in cells expressing different MC4R variants. The cAMP response to α-melanocyte stimulating hormone (α-MSH) and Setmelanotide was studied for wildtype (WT) and 17 mutant forms of MC4R; mean EC50 (concentration required to elicit 50% of the maximal response) of experiments performed in duplicate with 11 point dose titrations. Some variants (R18C, V50M, I102T, G231S, R305W) have been studied previously and values were taken from the relevant reference [42]. The ability of Setmelanotide to rescue signaling by MC4R mutants was calculated (EC50 of α-MSH/EC50 of Setmelanotide) and varied considerably between mutants. The ratio could not be precisely determined for some variants (G55D, E61K, C271Y) because of the high concentrations of α-MSH needed.

Using this functional classification, we examined phenotypic data from 224 children and 264 adults carrying *MC4R* mutations. We found that *MC4R* variant homozygotes were significantly more obese than heterozygotes (p < 0.001); there was no significant difference in BMI between those carrying heterozygous complete vs partial LOF variants (Figure 1E). We did not identify significant genotype–phenotype correlations for most phenotypes after adjusting for age, gender, and BMI (data not shown). Of interest, we found that children heterozygous for complete LOF variants consumed slightly more at an *ad libitum* test meal (mean 121.1 kJ/kg lean mass ± SEM 12.3) than those with partial LOF mutations (mean 82.2 kJ/kg of lean mass ± SEM 10.4), although this was not statistically significant (p = 0.07, Figure 1F). This effect was more pronounced when analyzing the data for children under 10 years of age (mean 154.7 kJ/kg lean mass ± SEM 13.2 in complete LOF, 96.6 ± 15.2 in partial LOF; p = 0.02, Figure 1G). One possible explanation is that young children may be less influenced by the presence of observers in the research setting. There was a similar trend for homozygous carriers of *MC4R* variants but with limited statistical power due to the small number of individuals. These findings support the notion that variation in melanocortin tone is a key modulator of human energy intake.

### 3.2 Setmelanotide rescues signaling by MC4R mutants *in vitro*

As MC4R is known to be a Gα_s_-coupled GPCR [30], we studied the ability of increasing agonist concentrations to induce cyclic AMP (cAMP) in transiently transfected cells expressing WT or mutant MC4Rs, measuring the maximal response (E_max_) and the agonist potency (EC_50_). We have previously shown that Setmelanotide exhibits nanomolar potency in a cAMP accumulation assay (EC_50_ = 0.27 nM) [21]. In our cell based reporter system Setmelanotide is about 10–20 fold more potent than the endogenous ligand α-MSH at WT MC4R. Here, we tested the ability of Setmelanotide to rescue a randomly selected group of published MC4R variants (Figure 2). We found that Setmelanotide was significantly more potent than α-MSH for all tested variants. However, there was considerable variation in the magnitude of the rescue response (defined as EC_50_ α-MSH/EC_50_ Setmelanotide) with Setmelanotide being up to 500-fold more potent than αMSH at some mutant MC4Rs (e.g. E61K and G55D) (Figure 2).

### 3.3 Setmelanotide administration in MC4R null and MC4R heterozygote null mice

**Figure 3:**
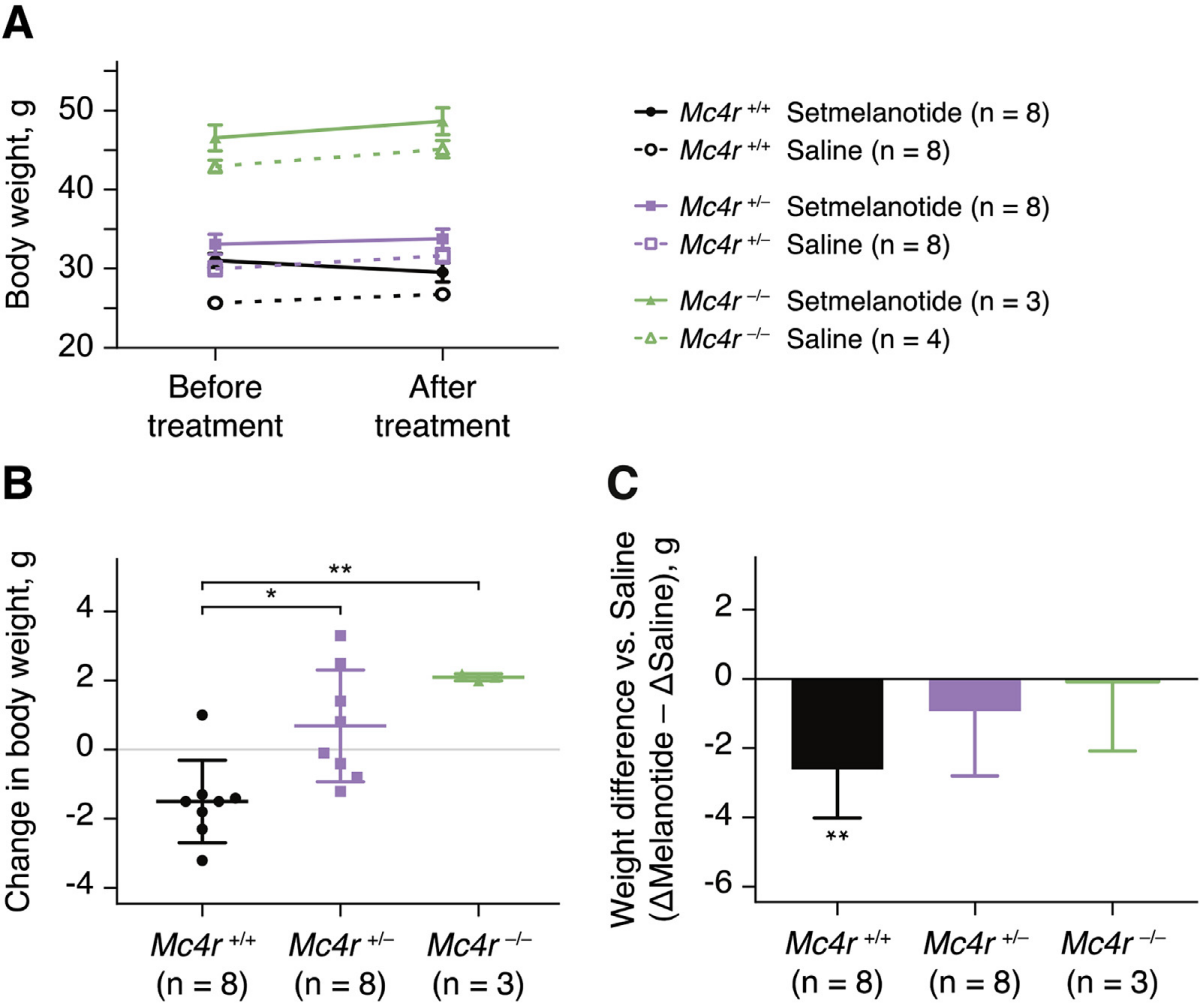
**Effect of Setmelanotide administration in mice lacking Mc4r.***Mc4r*^+/+^, ^+/−^, and ^−/−^ mice were infused with 1200 nmol/kg/day of Setmelanotide, or vehicle (saline), using an implanted subcutaneous minipump. (A) Body weight before (day 0) and after (day 9) among all treatment groups. (B) Cumulative 9-day weight change among all Setmelanotide treated groups. Each symbol represents a single test animal's change in body weight. Statistical significance between groups is calculated using one-way ANOVA with Bonferroni post-test. (C) Setmelanotide mediated 9-day weight improvement over saline treated groups. These values are calculated by subtracting the average body weight change in saline treated groups from the average body weight change in Setmelanotide treated groups (ΔSetmelanotide − ΔSaline). A treatment that causes no change compared to saline would be represented by a weight difference of zero. Values displayed are Mean +/− SD and statistical significance is calculated using a T-test versus saline controls. *P < 0.05; **P < 0.01.

We studied the effect of Setmelanotide administration in mice lacking *Mc4r*. Wild-type (*Mc4r* ^+/+^), heterozygous null (*Mc4r* ^+/−^), and a small number of homozygous null (*Mc4r* ^−/−^) mice were placed on a high fat diet (45% kcal fat) and then implanted with subcutaneous osmotic pumps containing either 1200 nmol/kg/day Setmelanotide or vehicle (saline); body weights were monitored for 9 days. All saline treated groups gained weight in response to the high fat diet. *Mc4r* ^+/+^ mice treated with Setmelanotide exhibited weight loss, while *Mc4r* ^−/−^ mice treated under the same conditions, gained a comparable amount of weight to their vehicle-treated controls. Setmelanotide-mediated weight loss seemed to be mediated by a reduction in food intake, rather than changes in activity or energy expenditure (Figure S3), although the magnitude of the effect was small in these studies. Interestingly, *Mc4r*^+/-^ mice exhibited an intermediate response to Setmelanotide treatment as evidenced: i) by a reduction in absolute weight gain over the period (Figure 3A); ii) when comparing weight gain to baseline in treated mice (Figure 3B); and iii) the Setmelanotide-mediated improvement in body weight over saline (Figure 3C) (the latter corrects for the weight gain seen in vehicle-treated mice on the high fat diet). Overall, it is apparent that *Mc4r* ^+/+^ and ^+/−^ mice lose weight with Setmelanotide, with wild-type mice being the most sensitive to treatment, while *Mc4r* ^−/−^ mice do not respond (as also shown previously [22]).

### 3.4 Clinical response to Setmelanotide: a phase 1b trial in MC4R deficiency

We examined the effects of Setmelanotide on weight loss in randomized, double blind placebo controlled Phase 1b trials. A total of 49 healthy obese (BMI 26.2–40.3 kg/m^2^) individuals between the ages of 19–53 years (41 males and 8 females) were enrolled in 5 cohorts and randomized to treatment with either Setmelanotide or placebo for 14 or 28 days via continuous subcutaneous infusion. Generally, each cohort enrolled nine individuals, six active and three placebo. *MC4R* was sequenced in all participants; one subject was identified as heterozygous for a predicted partial loss of function variant (G252S), whereas all other individuals had a normal coding sequence and were thus referred to as Obese Controls. A sixth cohort of healthy obese (BMI 31.5–51.7 kg/m^2^) people between the ages of 22–57 years (2 males and 6 females) known to be heterozygous for complete or partial loss of function mutations in *MC4R* were enrolled in the trial and received 0.01 mg/kg/24 h for 28 days. The previously identified subject (G252S) from Cohort 4 was enrolled in this MC4R heterozygote cohort (Table 1).

**Table 1:** Changes in weight and waist circumference with Setmelanotide in heterozygous MC4R deficiency and obese controls.

Treatment arm	Heterozygous MC4R deficiency				Obese controls			
	Baseline, mean ± SD	Day 29, mean ± SD	Change from baseline, mean (95% CI)	P-value	Baseline, mean ± SD	Day 29, mean ± SD	Change from baseline, mean (95% CI)	P-value
**Weight, kg**								
Setmelanotide	130.46 ± 22.96	126.98 ± 22.96	−3.48 (−4.99, −1.96)	<0.0001	97.86 ± 17.18	94.79 ± 16.50	−3.07 (−4.11, −2.04)	<0.0001
Placebo	101.75 ± 21.71	100.90 ± 23.29	−0.85 (−3.48, 1.78)	0.52	98.26 ± 7.99	99.16 ± 7.30	0.90 (−0.44, 2.24)	0.18
Comparison of Setmelanotide vs. placebo			−2.63 (−5.66, 0.41)	0.09	–		−3.97 (−5.67, −2.28)	<0.0001
**Waist circumference, cm**								
Setmelanotide	132.8 ± 18.1	127.0 ± 17.4	−5.83 (−9.70, −1.96)	0.005	107.2 ± 8.9	105.4 ± 8.5	−1.80 (−2.72, −0.88)	0.0004
Placebo	112.3 ± 13.1	111.5 ± 13.4	−0.75 (−7.45, 5.95)	0.82	112.3 ± 12.4	113.0 ± 13.0	0.67 (−0.52, 1.85)	0.26
Comparison of Setmelanotide vs. placebo			−5.08 (−12.82, 2.66)	0.19	–		−2.47 (−3.96, −0.97)	0.002

Randomized, double blind placebo controlled trial of Setmelanotide subcutaneous infusion 0.01 mg/kg/day vs placebo for 28 days in heterozygous carriers of melanocortin 4 receptor mutations (*MC4R*, n = 6 on Setmelanotide and n = 2 on placebo list of variants in Table S3) and obese controls (right section, 5 subjects on setmelanotide and 3 on placebo). Mean weight and waist circumference values (3 readings) were averaged and compared with ANOVA of change from baseline as the dependent variables and treatment, day and the interaction as factors.

Although the total number of individuals in each cohort of this Phase 1 trial was small, significant weight loss was observed in all Obese Control cohorts receiving ≥0.01 mg/kg/24 h of Setmelanotide compared to placebo (Table 1). In the MC4R mutation carriers, placebo subtracted group mean differences in weight loss (dose 0.01 mg/kg/24 h) of ∼0.6 kg/week were observed (Table S3). Although this result did not reach statistical significance (p = 0.09) when compared to placebo, weight loss from baseline was significantly reduced (p < 0.0001). Weight loss was accompanied by reductions in waist circumference (Table 1).

In view of the previous adverse effect profile of MC4R agonists, HR and BP were frequently monitored throughout the 14- to 28-day treatment periods for each dose, and for 24 h after completion of treatment. 24-hour ambulatory BP monitoring (ABPM) was performed on a baseline day, halfway through treatment and on the last dosing day of each cohort. There was no increase in HR or BP at any dose in either obese control or MC4R mutation carriers treated with Setmelanotide, as seen in other clinical studies with Setmelanotide. The adverse events (AEs) observed in this study were generally well-tolerated, mild, and intermittent. As is common in Phase 1 studies, AEs were extensively collected, and most of these AEs (except tanning) were also reported in the placebo group. AEs reported after repeated administration of Setmelanotide regardless of dose included: skin discoloration (tanning) (56.4%), headache (33.3%), skin lesions (17.9%), nausea (15.4%), penile erections (12.8%), infusion site pain (12.8%), and diarrhea (10.3%). In general, there were no differences in the frequency of these effects between the Obese Control and MC4R deficient groups. The most frequently reported adverse effect of Setmelanotide was skin tanning, which increased gradually at doses ≥0.01 mg/kg/24 h reaching a plateau by ∼2 weeks in most people. This effect was likely explained by activation of the closely related MC1R (Setmelanotide potency on MC1R = 5.8 nM). As well as clinical assessments of skin pigmentation, skin color was assessed quantitatively using a Mexameter. At baseline, individual inter-subject variability was high (reflecting ethnicities of participants); however, the intra-subject variability was low. For example, in one placebo group studied over 2 weeks, the baseline mean score for six individuals was 297.56 units and the 2-week post-dose (placebo) response was 290.22 units. In contrast, in the Setmelanotide treated Obese Controls, the baseline score in six individuals was 331.11 units, and the 2-week post-dose (active) value was 521.33 units, a result that was statistically significant (p < 0.0001) even in this small group of individuals. All patients were followed to resolution following active dosing (∼2–6 weeks).

## 4 Discussion

Our findings demonstrate that the melanocortin 4 receptor agonist, Setmelanotide can lead to weight loss in obese people with MC4R deficiency (on average 0.6 kg/week). Importantly, Setmelanotide induced weight loss over 4 weeks without eliciting an increase in HR or BP, in contrast to other MC4R agonists tested to date. Additional studies are needed to determine whether the data presented here are effective predictors of weight loss in larger studies enrolling cohorts of patients carrying complete and partial MC4R LOF variants. Such studies may also provide information about the mechanisms by which weight loss occurs. A potential mechanism that could contribute to the efficacy of Setmelanotide in such patients is a chaperone effect, whereby agonists (or antagonists) can rescue mutant forms of the MC4R by restoring their expression at the cell surface. A number of prior studies have also shown that a subset of *MC4R* variants appear to be rescued in this manner when studied in cells with distinct MC4R agonists [31], [32], [33].

In a head-to head comparison of Setmelanotide and α-MSH, we found that some variants were rescued more efficiently than others, especially those with a high mutant/wild type EC_50_ ratio. At present the structure of the MC4R has not been determined, although homology modeling on MC1R has been used to suggest possible structural explanations for the molecular mechanisms by which mutations result in a LOF [31]. Interestingly, we find an overrepresentation of non-synonymous variants in transmembrane domain (TM)-I, intracellular loop-1 (ICL-1), TM-II, the intracellular part of TM-VII, and helix 8, domains which form a so-called hydrogen bond network which has been demonstrated to play an important role in receptor activation and signaling specificity [34], [35]. There are surprisingly few LOF variants in the N-terminal domain and in extracellular segments of the inward looking parts of transmembrane domains which constitute the large binding pocket (TM-III, TM-IV, TM-V, TM-VI, TM-VII). Further studies will be needed to more fully characterize the mutants reported here and the mechanisms by which a subset are rescued by Setmelanotide.

Given the large number of possible MC4R variants, we were fortunate to find one patient enrolled who carried the heterozygous C271Y MC4R variant, which was earlier, independently classified as being disproportionally efficiently rescued by Setmelanotide in cells. Indeed, this patient was one among three complete LOF MC4R variant carriers treated with Setmelanotide who showed a profound weight loss response (0.9 kg/week). This could be a basis for future patient stratification whereby certain patients might benefit more readily from Setmelanotide treatment than others.

Possible reasons for the cardiovascular profile of Setmelanotide, compared to previous MC4R agonists, might be that some lead compounds tested to date differentially penetrate the CNS [36] and, at these sites, may couple through different G-protein signaling pathways. Recently, Weinstein and colleagues showed that disruption of melanocortin receptor Gα_s_ and Gα_q11_ signaling in the paraventricular nucleus of the hypothalamus, led to divergent phenotypes in rodents [37], and several studies have shown that MC4R signaling is more complex than previously thought including evidence of biased signaling [38] and evidence for diverse ligands exhibiting distinct temporal cellular signaling selectivity [39].

We have shown that individuals with MC4R deficiency as well as those expressing the wild type MC4R lose weight following Setmelanotide treatment. Current preclinical and clinical MC4R pathway deficiencies treated with Setmelanotide indeed predict an order of Setmelanotide treatment sensitivity where MC4R pathway upstream mutations (POMC) are more sensitive to Setmelanotide treatment than wild type genotypes [40], which appear, in turn, more responsive to Setmelanotide than most MC4R LOF variants. We anticipate that stratification of patients harboring mutations in the leptin-melanocortin pathway, which include the pro-opiomelanocortin, proprotein convertase subtilisin/kexin type 1, leptin receptor and defects in MC4R, will help targeting Setmelanotide to the genetic subgroups that can optimally respond to treatment with this promising drug candidate.

## Funding

This work was supported by the Wellcome Trust (I.S.F.), the National Institute for Health Research Cambridge Biomedical Research Centre (S.O'R., I.S.F.), the Bernard Wolfe Health Neuroscience Fund (I.S.F.), the European Research Council (I.S.F.), and the Swiss National Science Foundation (PBLAP3-145870, P3SMP3-155318, PZ00P3-167826 to T.-H.C.). Funds were also obtained from the Clinical Research Programs on Obesity (Assistance Publique-Hôpitaux de Paris, and the Direction of Clinical Research (CRC) (PHRC 02076 to K.C.), as well as the Institut Benjamin Delessert and the Fondation pour la Recherche Médicale and the National Agency of Research (program “Investissements d'Avenir” with the reference ANR-10-IAHU-05). The clinical trial was supported by Rhythm Pharmaceuticals.

## Author contributions

LVDP, ISF, and KC designed the study and wrote the paper with THC, AG and BD who led the functional classification and genotype–phenotype correlation analysis. HC, AG, SS, CF, BH, LS, KG, and LVDP designed and performed the clinical trial of Setmelanotide and analyzed the resulting data. JM and EMO performed the functional studies and analyzed the resulting data of UK patient mutations; JMK, EH, RB, SOR, and ISF recruited UK patients to the GOOS cohort, collected and analyzed the clinical data; BD, PT, CPB, JMO, FM, and KC recruited patients to the French cohort, collated and analyzed the clinical data. RA performed the functional analysis of the French mutations and JML, JLB, and DP the genetic screening in the French patients. BP and RC conducted the murine studies.
