# Supplementary material for: Evaluation of a melanocortin-4 receptor (MC4R) agonist (Setmelanotide) in MC4R deficiency
Source: Mol Metab. 2017 Jul 8;6(10):1321–9. doi: 10.1016/j.molmet.2017.06.015 (PMC5641599; doi:10.1016/j.molmet.2017.06.015)
Supplement: Supplementary file 1 [file mmc1.pdf]

Figure S1. Systematic review of published MC4R variants

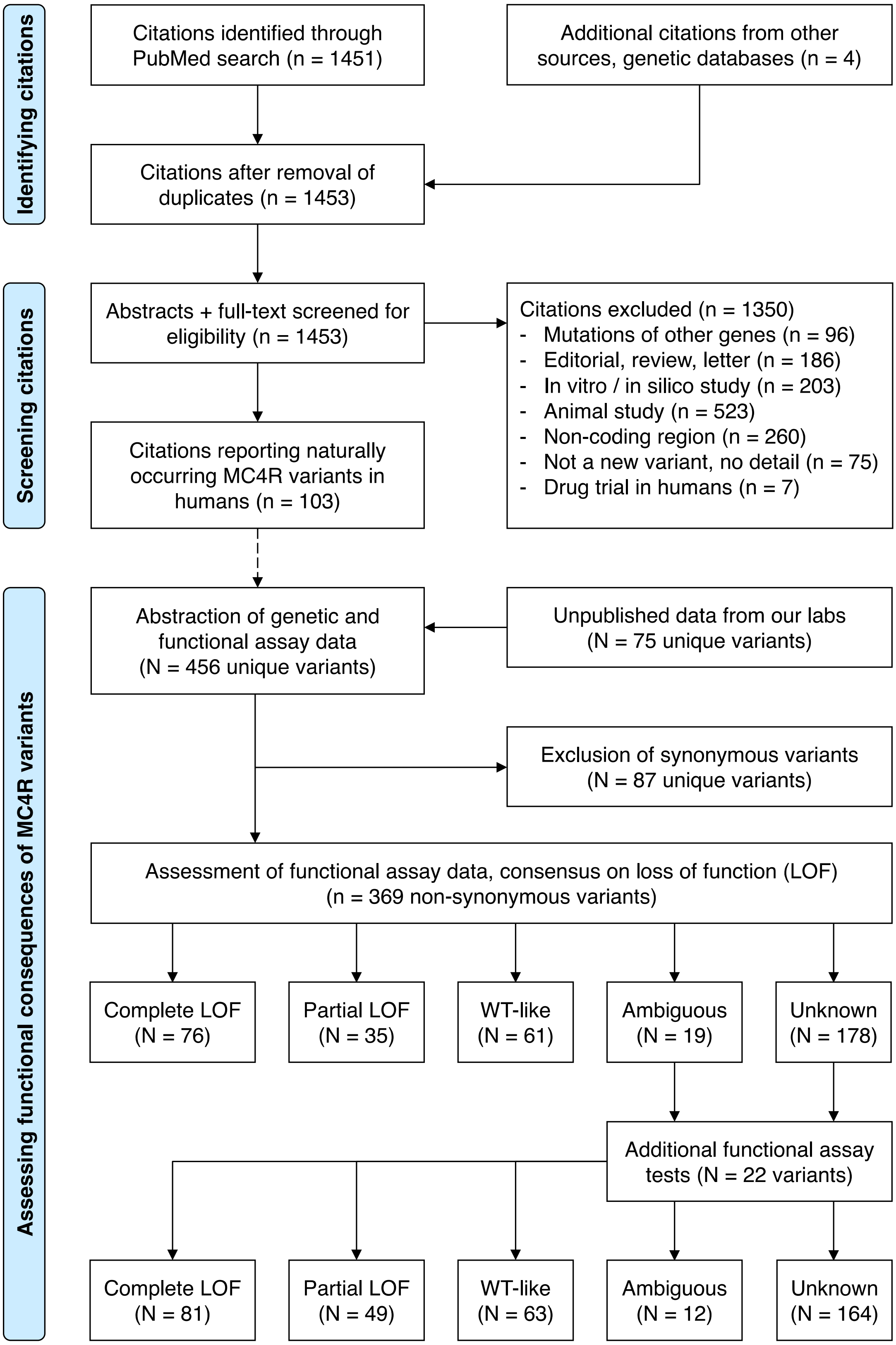

Legend:

The systematic review of the literature on melanocortin 4 receptor (MC4R) yielded 1451 publications and was conducted as shown. In addition, 75 unpublished mutations from UK and French cohorts were included. A total of 456 mutations were identified (**see Table S2**). All published and in-house functional assay data were collated and assessed to reach a loss of function (LOF) consensus, based on the criteria listed in **Figure S2** and reported as complete
LOF, partial LOF or wild type (WT)-like.

Figure S2. Interpretation of functional assay parameters for each ligand tested with MC4R variants

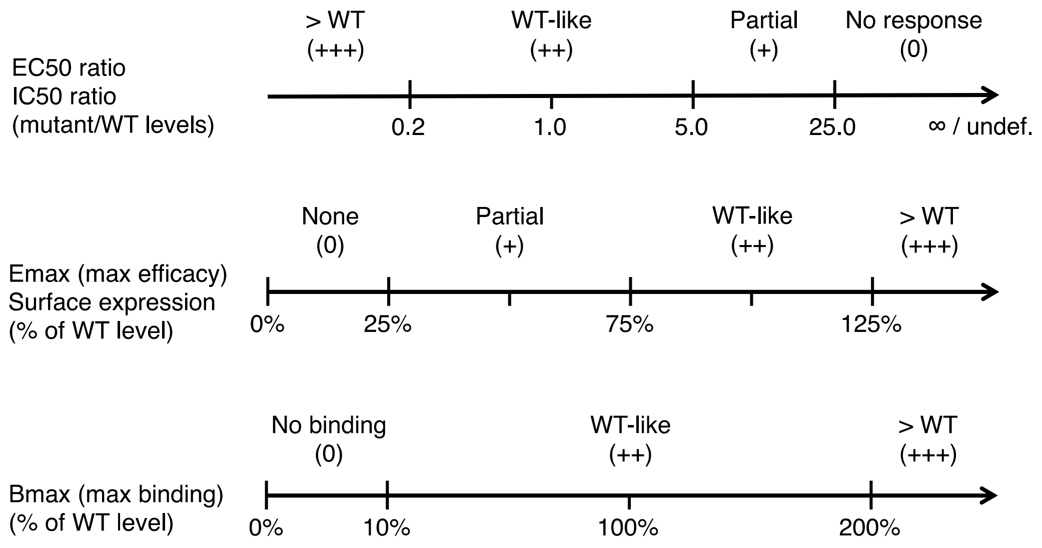

<u>Legend</u>:
Systematic review of literature of MC4R variants. The following criteria used to assess loss of function (LOF) of MC4R mutations. Functional assay data from the literature were collated. The cyclic AMP response to MC4R ligands for reported MC4R mutations (compared to wild type [WT] MC4R) was classified as shown; surface expression and ligand binding activity data were also collated.

Figure S3. Setmelanotide administration to mice.

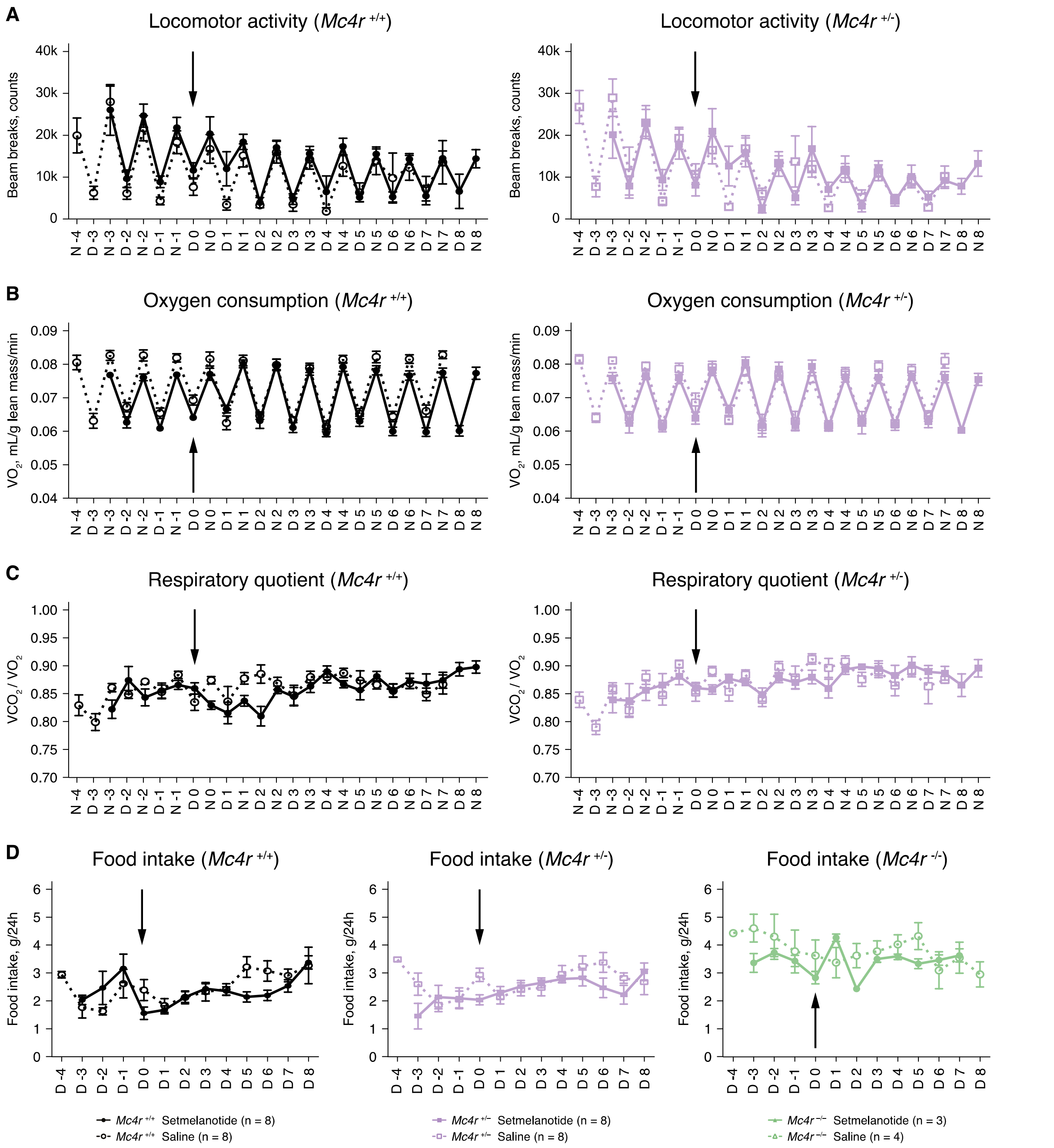

Legend: Setmelanotide treatment does not significantly affect locomotor activity or metabolic rate in treated mice. *Mc4r* $^{+/+}$, $^{+/-}$, and $^{-/-}$ mice were infused with 1200nmol/kg/day of Setmelanotide, or vehicle (saline), using an implanted subcutaneous minipump. The time of implantation is indicated using an arrow on each graph. (A). Daily locomotor activity as measured by beam breaks in Setmelanotide- or Saline-treated Mc4r $^{+/+}$ and $^{+/-}$ mice. (B). Oxygen consumption (VO_2) averaged every 12 hours according to light (Day) and dark (Night) cycles. (C). Respiratory quotient (VCO_2/VO_2) averaged every 12 hours. (D) Daily food intake (total) every 24 hours, as measured automatically by the Sable Promethion System in Mc4r $^{+/+}$ and $^{+/-}$ mice, or manually in *Mc4r* $^{-/-}$ mice.

Table S1. Functional characterization of MC4R mutations

MC4R variant	α-MSH						Surface expression (% of WT)			Functional status	
	EC_{50} (nM)			E_{max} (% of WT)		n					
	mean	SEM	F_{mut}	mean	SEM		mean	SEM	n	Based on literature	Updated with experimental data
WT	5.76	1.12	n/a	100%	n/a	8	100%	1.1%	13	n/a	n/a
E42K	7.54	1.78	1.3	96%	5.3%	4	114%	3.9%	3	unknown	WT-like
T53I	3.27	0.84	0.57	78%	2.7%	4	33%	2.1%	3	unknown	WT-like
N72S	12.2	5.38	2.1	52%	1.3%	4	79%	2.7%	3	unknown	pLOF
H76R [a]	1.06	0.17	0.18	61%	3.6%	3	77%	1.6%	3	ambiguous	pLOF
Y80C	37.3	9.63	6.5	38%	5.5%	4	54%	1.6%	3	unknown	pLOF
V95I [b]	17.1	5.11	3.0	47%	9.2%	4	4.3%	1.5%	4	ambiguous	pLOF
D122Y	n/a	n/a	n/a	0% [c]	n/a	4	31%	2.3%	3	unknown	cLOF
D126E	n/a	n/a	n/a	0% [c]	n/a	4	99%	5.3%	3	unknown	cLOF
S131R	n/a	n/a	n/a	0% [c]	n/a	4	35%	1.3%	3	unknown	cLOF
A144S [b]	12.3	3.14	2.1	33%	5.1%	4	und.	n/a	4	unknown	pLOF
D146G	12.3	1.66	2.1	61%	0.8%	4	85%	2.4%	3	unknown	pLOF
I170V	17.5	2.11	3.0	63%	6.8%	4	13%	1.5%	4	ambiguous	pLOF
A175T [b]	15.1	1.81	2.6	35%	3.5%	3	und.	n/a	4	ambiguous	pLOF
S180P	62.5	17.9	11	75%	2.9%	4	109%	1.5%	3	unknown	pLOF
G238D	13.4	0.67	2.3	78%	6.9%	4	105%	3.9%	4	WT-like	WT-like
G252S	36.4	9.62	6.3	56%	7.1%	3	22%	1.6%	3	ambiguous	pLOF

V253I	14.9	2.45	2.6	59%	12%	3	98%	4.1%	4	ambiguous	pLOF
P260T	n/a	n/a	n/a	0% [c]	n/a	4	und.	n/a	4	unknown	cLOF
I269N	n/a	n/a	n/a	0% [c]	n/a	4	und.	n/a	4	ambiguous	cLOF
F284L	n/a	n/a	n/a	42% [d]	n/a	6	103%	6.9%	3	unknown	pLOF
K314E	29.7	6.57	5.2	35%	7.7%	4	62%	2.8%	4	unknown	pLOF
R331K	9.61	1.96	1.7	70%	7.8%	4	59%	2.1%	4	unknown	pLOF

Footnotes: Related to Figure 1B-D. Twenty-two MC4R mutations, which were newly identified or for which existing published data were ambiguous or incomplete, were pharmacologically characterized with a CREB-luciferase reporter assay in transiently transfected HEK293 cells. Dose-response curves were used to determine potency of α-melanocyte stimulating hormone (α-MSH) for each mutant compared with the wild type-specific value, indicating the -fold shift (F_{mut}). Relative endogenous agonist efficacy was estimated through normalization of the dose response curves to maximal α-MSH-induced stimulation of wild type receptor (100%) and basal luciferase activity in mock-transfected cells (0%). Expression of each mutation was assessed by cell surface ELISA and is given as a fraction of the wild type receptor level. Functional classification based on the systematic review of the literature and after further pharmacological characterization is reported as complete loss of function (cLOF), partial loss of function (pLOF) or wild type (WT)-like. Data are represented as mean ± SEM, n/a – not applicable; – undetectable. [a] Variant exhibiting significant level of constitutive activity (mean 11 ± SEM 2.4%) as compared to wild type efficacy (E_{max}). No detectable constitutive activity was observed for MC4R wild type and other mutants. [b] Variants responding to stimulation with α-MSH (showing partial loss of function in terms of efficacy) despite no detectable surface expression. [c] Values assigned arbitrarily as no detectable response to stimulation with α-MSH was observed. [d] Maximal efficacy not assessed due to dose-response curve profile not reaching upper plateau level, value presented is a response (relative to wild type) observed for the highest dose of α-MSH (1µM).

Table S2. Functional classification dataset with MC4R functional assay data

See separate Excel spreadsheet

Table S3. Weight loss with Setmelanotide in heterozygous MC4R mutation carriers

Treatment arm	MC4R variant – loss of function	Weight loss on day 8, kg	Weight loss on day 15, kg	Weight loss on day 22, kg	Weight loss on day 29, kg
Setmelanotide	I269N – complete	3.4	4.3	5.0	5.9
Setmelanotide	C271Y – complete	1.9	3.3	4.0	3.6
Setmelanotide	Q307X – complete	3.5	3.5	4.5	5.0
Setmelanotide	R165Q – partial	2.7	2.2	3.0	5.0
Setmelanotide	R165W – partial	1.5	0.7	1.8	1.7
Setmelanotide	G252S – partial	1.5	1.1	1.9	0.9
Placebo	Q156X – complete	0.5	0.9	1.6	0.0
Placebo	G252S – partial	0.6	0.1	0.9	1.4

Footnotes: In a randomized, double blind placebo controlled trial of Setmelanotide subcutaneous infusion 0.01 mg/kg/day vs placebo for 28 days, heterozygous *MC4R* mutation carriers were weighed weekly from baseline. Setmelanotide led to a mean weight loss of 2.63 kg (SEM 1.50) compared to placebo (p=0.09).
